# Supplementary material for: Magnetic nanostickers for active control of interface-enhanced selective bioadhesion
Source: Nat Commun. 2025 Jul 10;16:6400. doi: 10.1038/s41467-025-61719-9 (PMC12246147; doi:10.1038/s41467-025-61719-9)
Supplement: Supplementary file 1 — Supplementary Information [file 41467_2025_61719_MOESM1_ESM.pdf]

Supplementary Information

Magnetic nanostickers for active control of interface-enhanced selective bioadhesion

Changshun Hou[1]*, Junjia Guo[2], Bonan Sun[1], Kai Fung Chan[2,3], Xin Song[1,4], Li Zhang[1,2]*

[1]Department of Mechanical and Automation Engineering, The Chinese University of Hong Kong; Shatin, N.T., Hong Kong, China.
[2]Department of Biomedical Engineering, The Chinese University of Hong Kong; Shatin, N.T., Hong Kong, China.
[3]Li Ka Shing Institute of Health Sciences, The Chinese University of Hong Kong; Shatin, N.T., Hong Kong, China.
[4]Department of Biomedical Engineering, City University of Hong Kong; Kowloon Tong, Kowloon, Hong Kong, China.

*Corresponding author. Email: changshou2@cuhk.edu.hk; lizhang@mae.cuhk.edu.hk

The PDF file includes:

Supplementary Text
Supplementary Figures 1 to 27
Supplementary Tables 1 to 2
Supplementary References

Supplementary Text

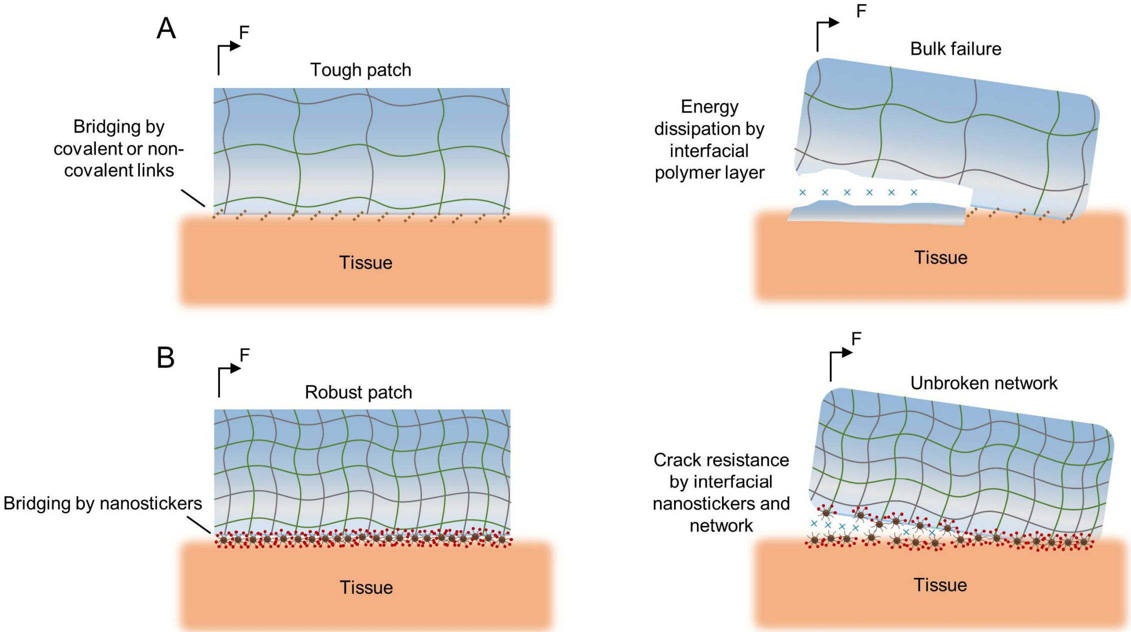

Supplementary Fig. 1. Comparison of conventional energy dissipation strategy and interface-enhanced strategy. (A) Conventional strategy relies on large energy dissipation by the bridged interfacial polymer layer. (B) Interface-enhanced strategy can integrate energy dissipation with great interfacial enhancement through the layer of anchored nanostickers and robust patch.

Supplementary notes: For conventional strategy, the toughness of interfacial polymer layer is increased as the patch is tough. The strategy focuses on improving the dissipated mechanical energy τ_D; however, the scarce functional groups and external barriers on tissue surfaces will tremendously decrease the intrinsic work of adhesion τ_0, which weakens the anchoring effect of bridged polymer layer. The decreased τ_0 will also impede the effective deformation and mechanical dissipation that are generated from the interface and the patch, resulting in a low τ_D.

For interface-enhanced strategy, the anchored nanostickers between the tissues and robust patch will significantly improve the interfacial strength for high τ_0. The highly cohesive nanostickers ascribed to abundant interparticle interactions such as strong magnetic force further provide stress transmission for peeling and resist the crack formation at the interfacial patch layer for high τ_D.

As adhesion energy relates to both τ_0 and τ_D[1], and τ_D can be influenced by τ_0, the efficient strategy to achieve robust bioadhesion should take both into account.

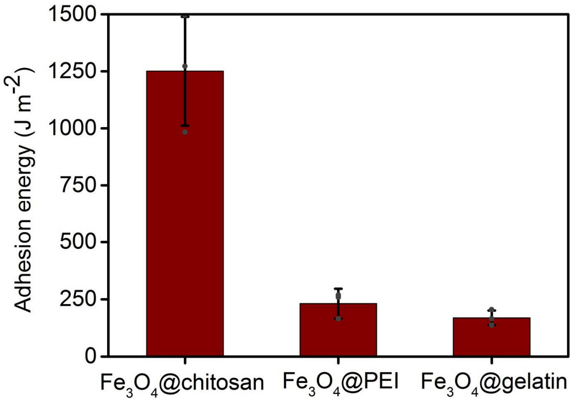

Supplementary Fig. 2. Adhesion energy is measured from pig skin-patch hybrids anchored with different nanostickers. Different cationic polymer-coated nanostickers (area density: 4 μg/mm^2) between the skin and PAAM-Alg patch can all improve the adhesion energy. Data are presented as mean ± SD; *n* = 3 independent samples.

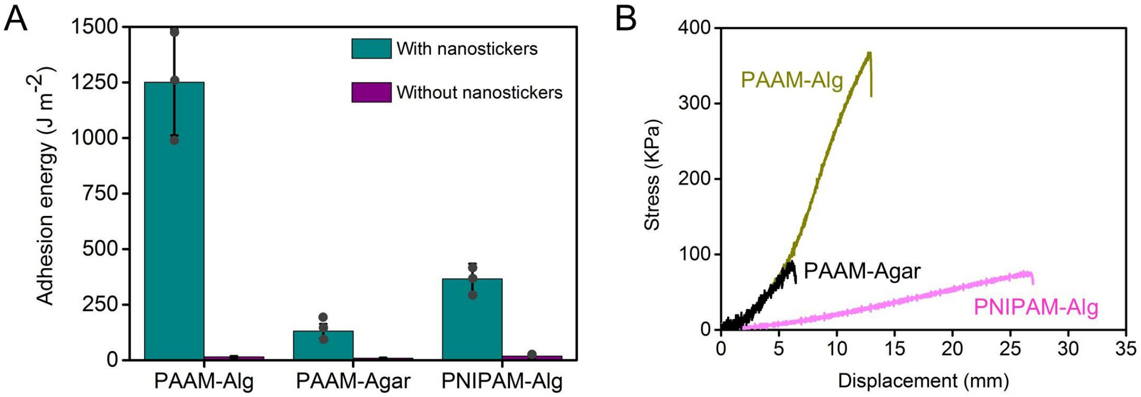

Supplementary Fig. 3. Adhesion energy is measured from hydrogel patches with various mechanical properties. (A) Adhesion energy can be improved by magnetic control of Fe_3O_4@chitosan nanostickers (area density: 4 μg/mm^2) for anchoring with various hydrogel patches on pig skin. Data are presented as mean ± SD; *n* = 3 independent samples. (B) Stress-displacement curves compare the mechanical properties of various hydrogel patches.

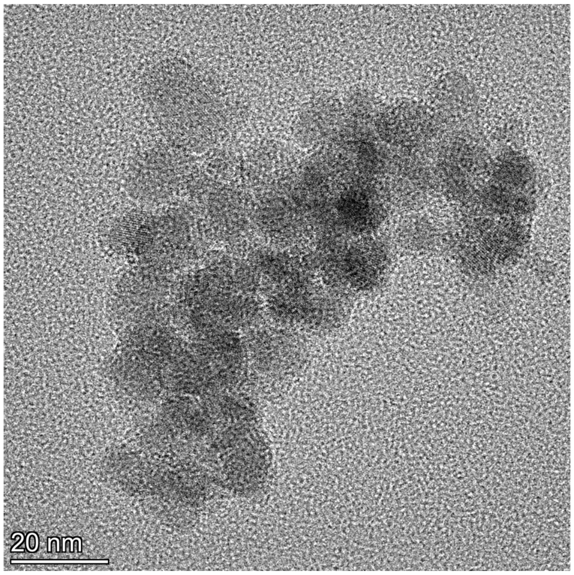

Supplementary Fig. 4. TEM imaging of prepared Fe_3O_4@chitosan nanostickers.

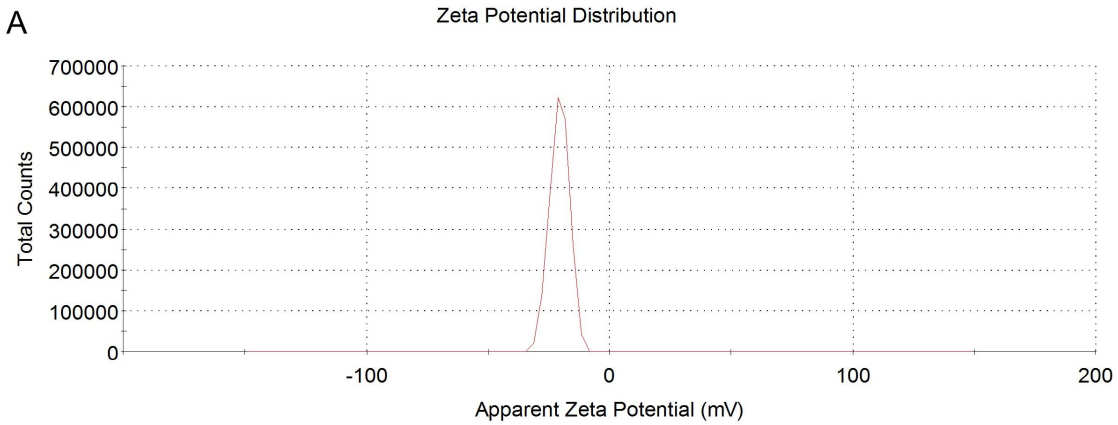

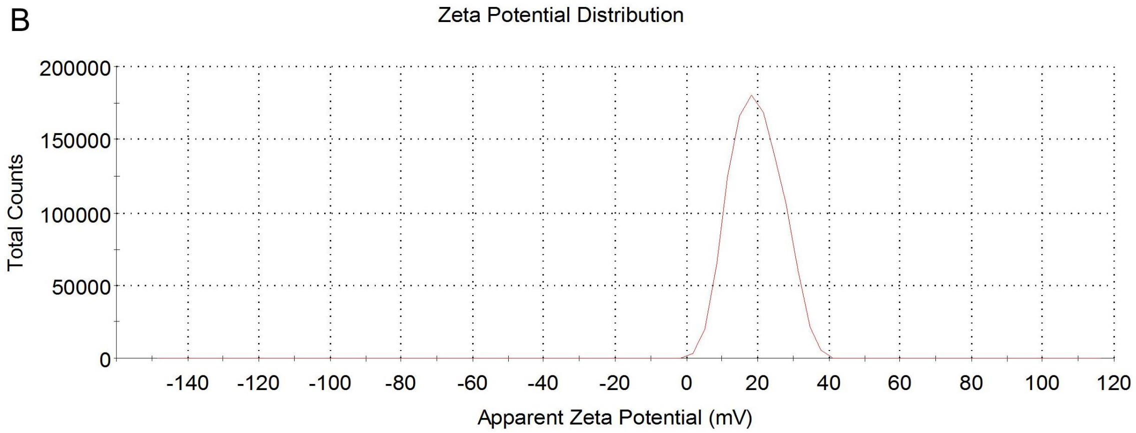

Supplementary Fig. 5. Zeta potential distributions of superparamagnetic particles and Fe_3O_4@chitosan. (A) Superparamagnetic particles show a negative surface potential

averaged at -20.6 mV. (B) Fe_3O_4@chitosan nanostickers show a positive surface potential at +19.6 mV.

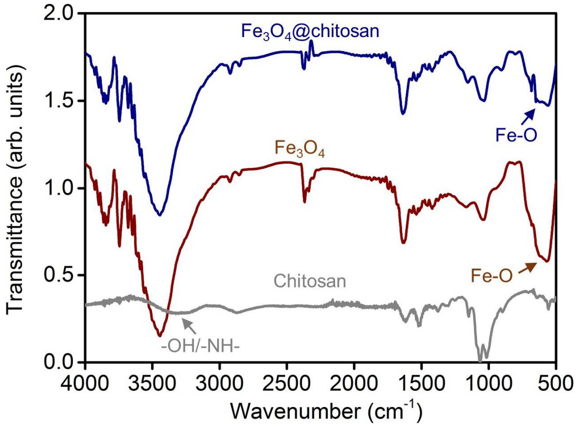

Supplementary Fig. 6. FTIR spectra of the main components in Fe_3O_4@chitosan. Characteristic peaks generated in Fe_3O_4@chitosan indicate that the surface functionalization of superparamagnetic particles do not change their chemical properties.

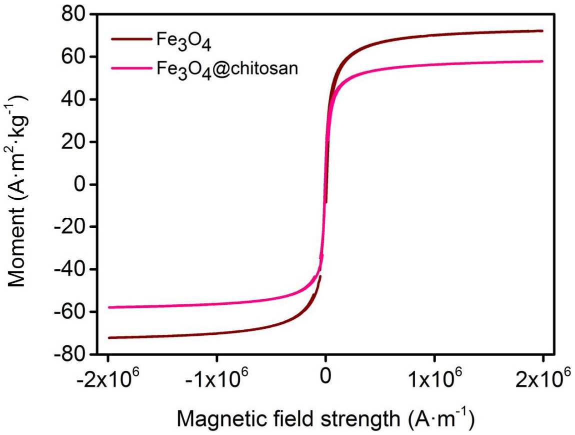

Supplementary Fig. 7. Magnetic hysteresis curves of superparamagnetic particles and Fe_3O_4@chitosan. Magnetic saturation of Fe_3O_4@chitosan shows a stable superparamagnetic property, which is close to that of superparamagnetic particles.

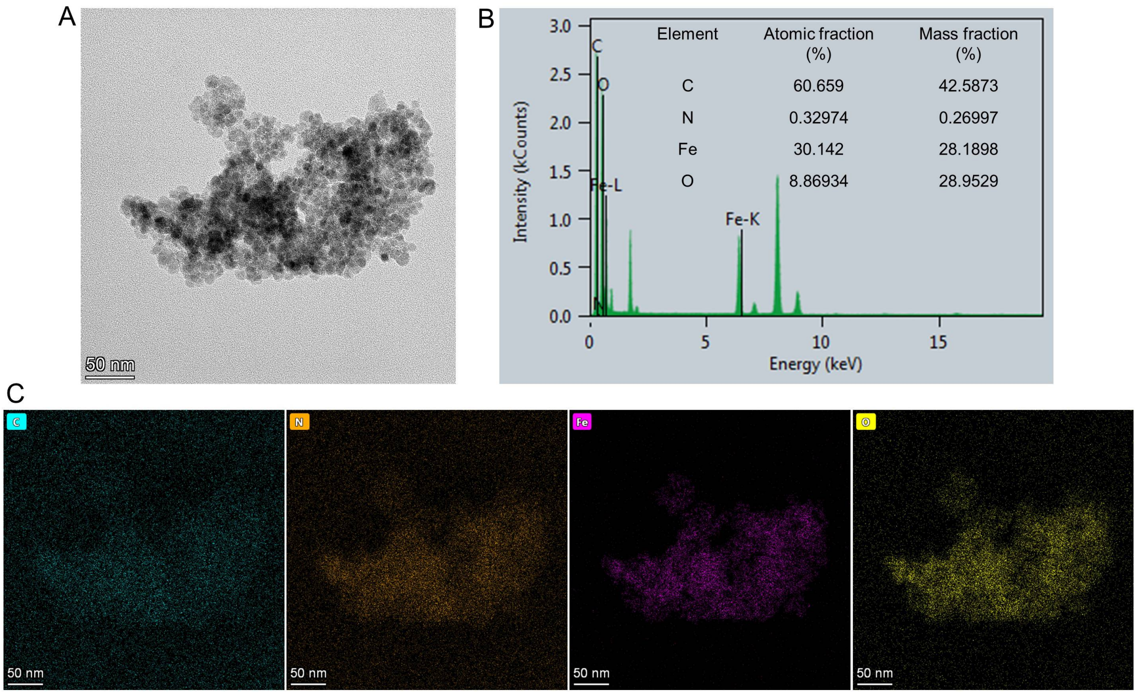

Supplementary Fig. 8. TEM imaging of Fe_3O_4@chitosan nanostickers after magnetic attraction. (A) Fe_3O_4@chitosan nanostickers show a dense aggregation under a magnet, suggesting the promotion of high cohesive force. (B) EDS analysis of the element content in Fe_3O_4@chitosan nanostickers. (C) Element mapping suggests that chitosan is well functionalized on Fe_3O_4 nanoparticles.

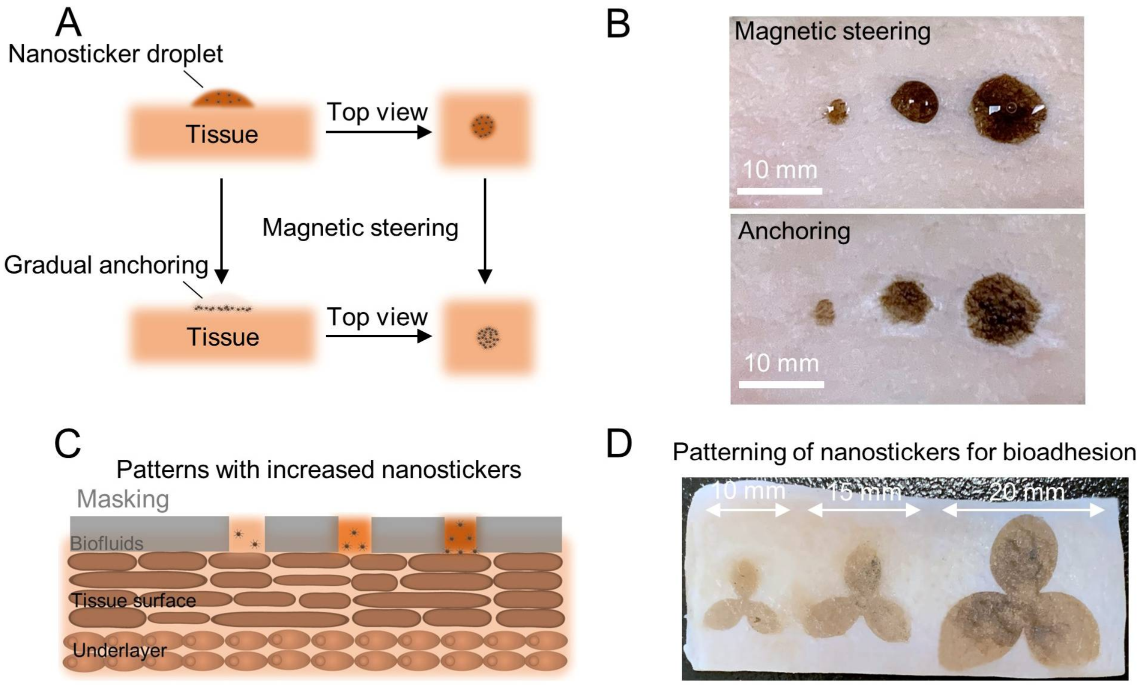

Supplementary Fig. 9. Nanostickers can be spatially anchored with different patterns. (A) Nanostickers can be directly anchored on tissues by remote magnetic field for some simple patterns such as circle. (B) Nanostickers can be anchored on pig skin within a circle by magnetic steering without using masks. (C) For complex patterns on tissues, nanostickers should be anchored with the help of masks. (D) Clover-shaped patterns with different resolutions are formed by the remote magnetic field and mask-assisted anchoring.

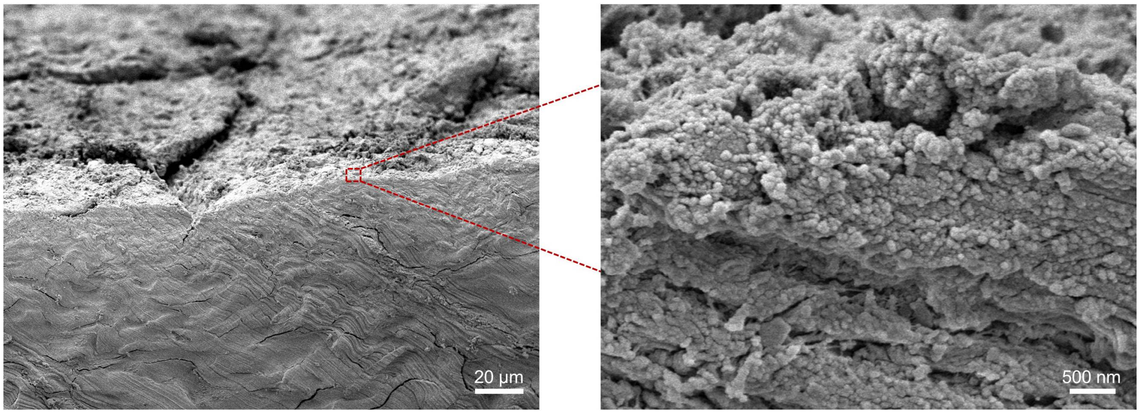

Supplementary Fig. 10. SEM images of the lyophilized pig skin. Nanostickers are densely aggregated and tightly anchored on the skin surface.

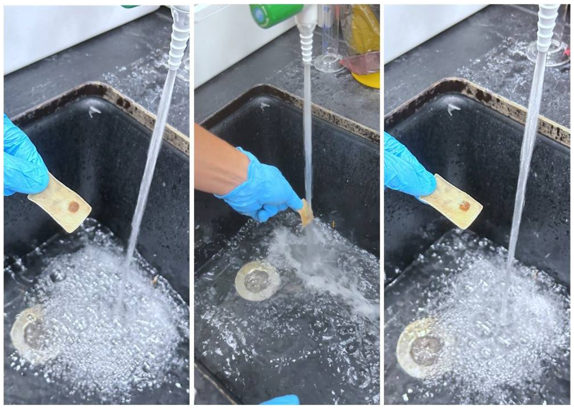

Supplementary Fig. 11. Spatially anchored nanostickers for water blasting test. Nanostickers are directly steered for anchoring on pig skin without masking. The anchored nanostickers can withstand the water blasting (2 bar) for 1 min without obvious damage.

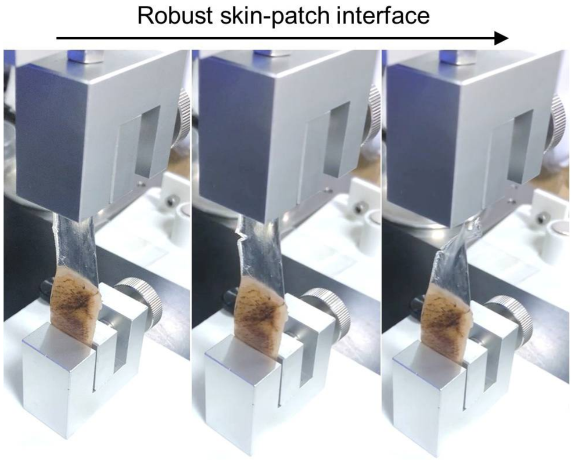

Supplementary Fig. 12. Tensile test of the nanostickers-anchored skin-patch hybrid. The interfacial strength between the pig skin and patch is stronger than the mechanical strength of the robust patch (anchored area: 2 cm of width × 2.5 cm of length).

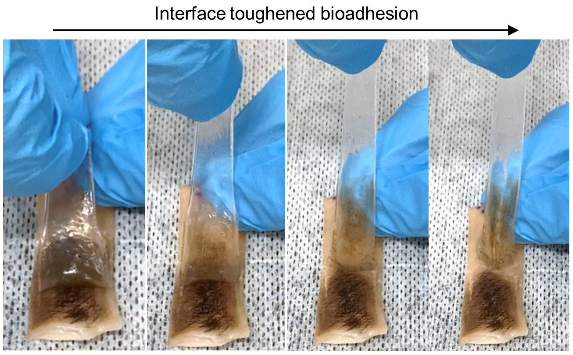

Supplementary Fig. 13. Peeling the patch from the anchored skin-patch hybrid. The anchored skin-patch interface is so robust that makes the patch to extend with a large deformation. Furthermore, most nanostickers remain anchored on skin upon peeling.

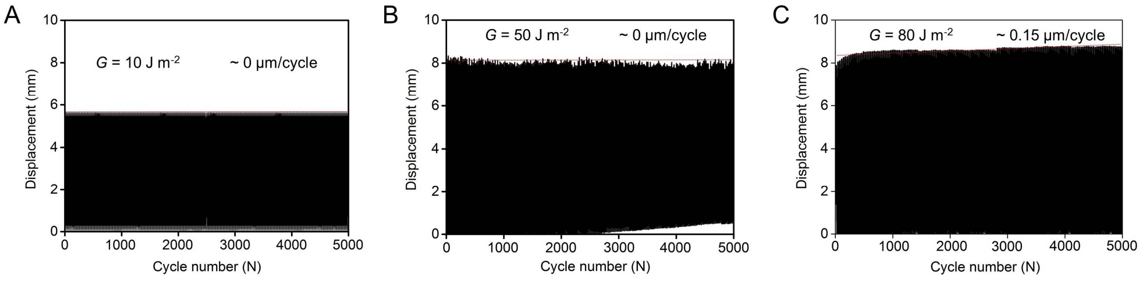

Supplementary Fig. 14. Representative cyclic peel curves for investigating fatigue resistant ability. Crack extension for each cycle d_c/d_N at different energy release rates G: (A) 10 J m^{-2}, (B) 50 J m^{-2}, and (C) 80 J m^{-2}.

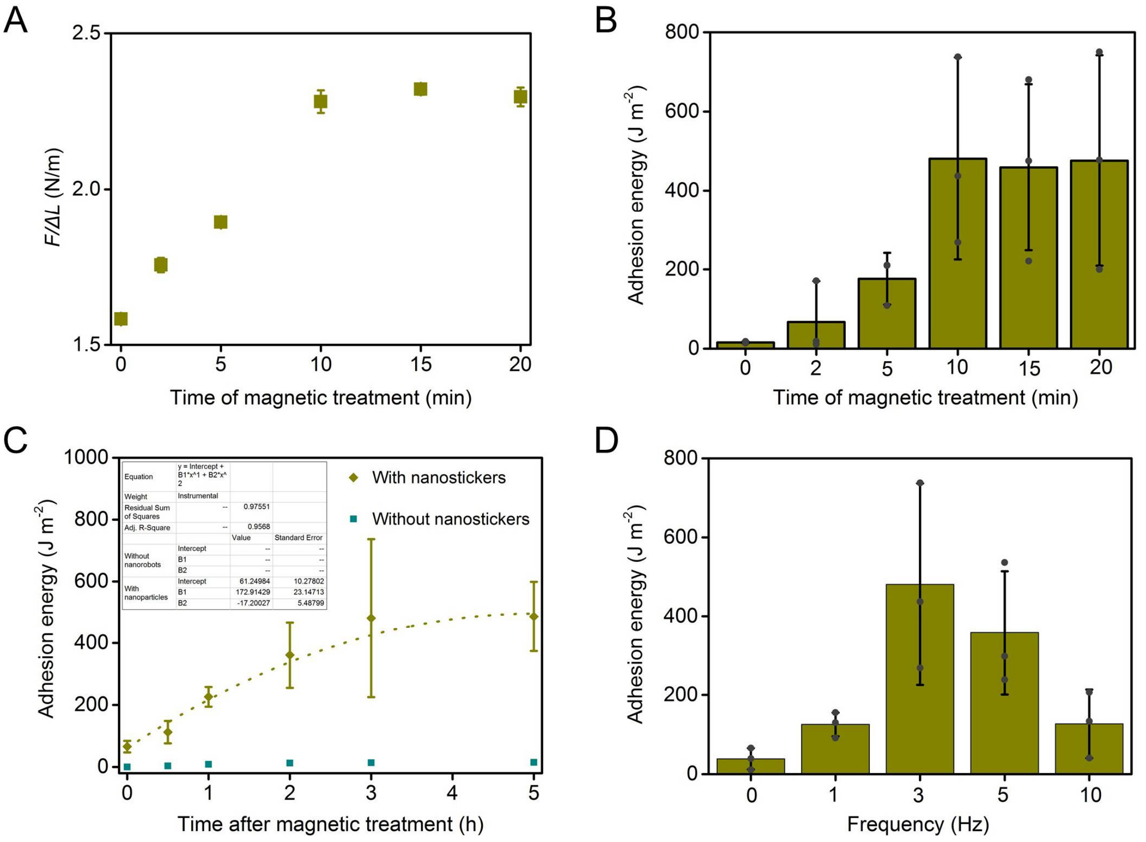

Supplementary Fig. 15. Adhesion energy is controlled with different magnetic parameters. (A) Work of retraction ($F/\Delta L$) varies with different time of magnetic steering of nanostickers on pig skin. All values are presented as mean ± SD for $n = 3$ independent experiments. (B) Adhesion energy of attached hydrogel patch varies with different time of magnetic steering of nanostickers (area density: 2 μg/mm^2) on pig skin. Data are presented as mean ± SD; $n = 3$ independent samples. (C) Adhesion energy is increased after magnetic treatment. Experimental data indicate that adhesion energy after 3 h of magnetic treatment can reach the peak and is close to that after 5 h of magnetic treatment. Fitted data with a polynomial implies the variation trend: $\tau = -17.2t^2 + 172.91t + 61.25 = -17.2(t-5.03)^2+495.83$; t is the time after magnetic treatment and τ represents the adhesion energy at t. Fitted data show that the adhesion energy at 3 h is approaching to the adhesion energy at 5 h. All values are presented as mean ± SD for $n = 3$ independent experiments. (D) Adhesion energy can be controlled by frequency of rotating magnetic field. Data are presented as mean ± SD; $n = 3$ independent samples.

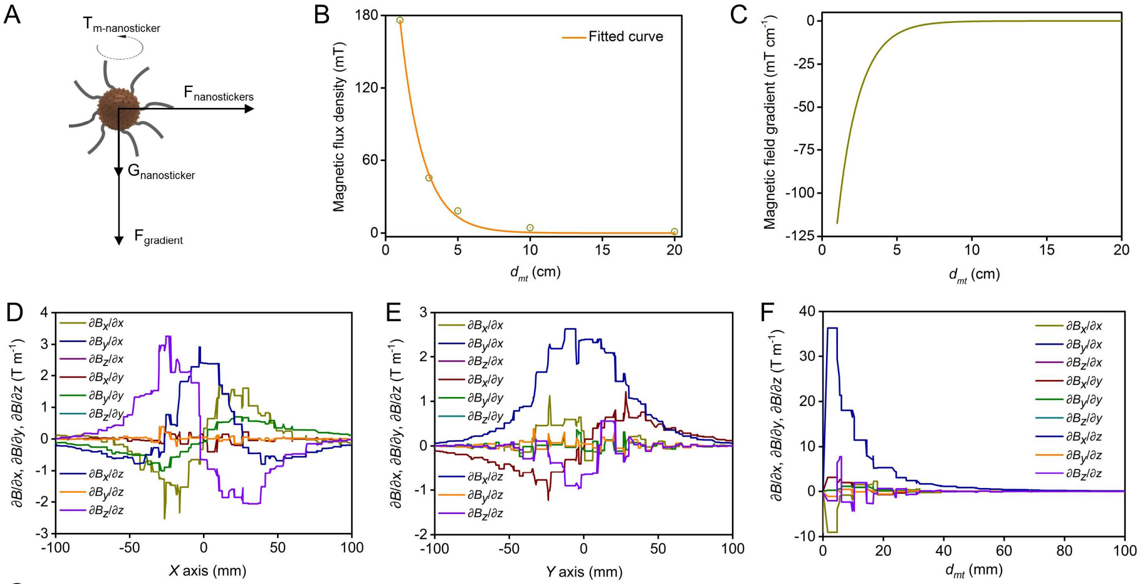

G

d_{mt} = 3 cm	$\partial B_x/\partial x$	$\partial B_y/\partial x$	$\partial B_z/\partial x$	$\partial B_x/\partial y$	$\partial B_y/\partial y$	$\partial B_z/\partial y$	$\partial B_x/\partial z$	$\partial B_y/\partial z$	$\partial B_z/\partial z$
Unit (T m^{-1})	0.33787	-0.05337	2.38861	-0.05337	-0.07897	0.06336	2.38861	0.06336	-0.65469

Supplementary Fig. 16. Magnetic force and torque analysis. (A) The main driving power of a single nanosticker for motion and anchoring is divided into four parts: gradient magnetic force $\mathbf{F}_{\mathbf{gradient}}$, magnetic torque $\mathbf{T}_{\mathbf{m\text{-}nanosticker}}$, nanosticker interactions $\mathbf{F}_{\mathbf{nanostickers}}$ (include magnetic force between nanostickers $\mathbf{F}_{\mathbf{m\text{-}nanostickers}}$ and intermolecular interactions $\mathbf{F}_{\mathbf{intermolecule}}$), and gravity $\mathbf{G}_{\mathbf{nanosticker}}$. (B) The magnetic flux density versus vertical distance d_{mt}. The magnetic field gradient at d_{mt} = 3 cm is extracted to -3.94 T m^{-1}. Dots represent the magnetic flux densities at different d_{mt}. (C) The magnetic field gradient at different d_{mt} can be obtained by calculating the variation of the magnetic flux density versus d_{mt}. All components of the magnetic flux density along (D) *X* axis and (E) *Y* axis at d_{mt} = 3 cm. (F) All components of the magnetic flux density for *Z*-axis points. (G) Concrete values for the components of magnetic flux density at a specific *Z*-axis point (d_{mt} = 3 cm). The components show a high rotational symmetry that indicate the $\nabla \times \mathbf{B}$ is zero at that point.

Supplementary notes: The force and torque are estimated for a nanosticker standing vertically above the center of magnet[2,3]. Because the nanosticker (~10 nm) is quite small compared to the spatial variation of the external magnetic field, the nanosticker is considered as a dot and thus **M** (the total dipole moment of the controlled nanosticker) can be regarded as a constant vector ($A \cdot m^2 \cdot kg^{-1}$). **B** represents the flux density of the external field at the integral point (T) and $m_{nanosticker}$ represents the mass of the nanosticker (kg). All components of **B** at different coordinates can be extracted from the above figures.

In a real condition, the nanostickers were rotated around *Z* axis by a spherical magnet (magnetization direction along the *XY* plane) at a low rotational frequency of 3 Hz (3

revolutions per second), and thus $\nabla \times \mathbf{B}$ could be described by one of Maxwell's equations (Ampère-Maxwell Law): $\nabla \times \mathbf{B} = \mu_0 (\mathbf{J} + \varepsilon_0 \frac{\partial \mathbf{E}}{\partial t})$. In this equation, μ_0 (vacuum permeability) and ε_0 (vacuum permittivity) are constants, **J** represents the current density, and **E** represents the electric field intensity, which are two variables in a certain environment. **J** can be decomposed into free current density ($\mathbf{J}_{free}$) and bound current density ($\mathbf{J}_{bound}$), and $\mathbf{J}_{bound} = \nabla \times \mathbf{M}$ (**M** represents the magnetization of materials), making $\nabla \times \mathbf{B} = \mu_0 (\mathbf{J}_{free} + \nabla \times \mathbf{M} + \varepsilon_0 \frac{\partial \mathbf{E}}{\partial t})$. From this perspective, the first term $\mathbf{J}_{free}$ is 0 because free current is existed in macroscopic conductors with external electric power, but the nanostickers have poor electrical conductivity (coated with insulating chitosan) and there was no supplied electric power to the nanostickers; the second term $\nabla \times \mathbf{M}$ is also 0 because **M** is regarded as a constant vector based on the fact that the nanosticker around 10 nm is quite small compared to the spatial variation of the external magnetic field; the third term $\varepsilon_0 \frac{\partial \mathbf{E}}{\partial t}$ equals to 0 or extremely closes to 0 due to the poor electrical conductivity of nanostickers (coated with insulating chitosan), low rotational frequency (3 revolutions per second) of magnet, and quite small volume of nanostickers (~10 nm) compared to the magnetic field in space, which are all enormous barriers to the generation of eddy current. In short, $\nabla \times \mathbf{B}$ equals or extremely approaches to 0 is rational in the experimental settings in terms of the above three terms, and therefore we can consider that the formula of magnetic force $\nabla (\mathbf{M} \cdot \mathbf{B})$ is also equivalent or extremely close to $(\mathbf{M} \cdot \nabla) \mathbf{B}$ in a real condition.

As a typical example, according to the magnetic flux density at d_{mt} = 3 cm (Fig. 3B and 3C) and magnetization curve in Supplementary Fig. 7, $|\mathbf{B}|$ is equal to 0.045 (T) and $|\mathbf{M}|$ can be calculated to 35 ($\mathrm{A \cdot m^2 \cdot kg^{-1}}$), $\mathbf{F_{gradient}}$ and $\mathbf{T_{m\text{-}nanosticker}}$ at a specific *Z*-axis point (d_{mt} = 3 cm) can be formulated as following:

$$\mathbf{F_{gradient}} = \nabla (\mathbf{M} \cdot \mathbf{B}) = (\mathbf{M} \cdot \nabla) \mathbf{B} + \mathbf{M} \times (\nabla \times \mathbf{B}) = (\mathbf{M} \cdot \nabla) \mathbf{B} = \mathbf{M} \cdot \nabla\mathbf{B};$$

$$|\mathbf{F_{gradient}}| = 35 \times 3.94 m_{nanosticker} = 138 m_{nanosticker}\ \mathrm{(N)};$$

$$\mathbf{T_{m\text{-}nanosticker}} = \int_{V_{nanosticker}} \mathbf{M} \times \mathbf{B}\ \mathrm{d}V_{nanosticker} = \mathbf{M} \times \mathbf{B};$$

$$|\mathbf{T_{m\text{-}nanosticker}}| = 0.045 \times 35 m_{nanosticker} = 1.58 m_{nanosticker}\ \mathrm{(N \cdot m)};$$

$\mathbf{F_{nanostickers}}$ can be formulated as $\mathbf{F_{nanostickers}} = \mathbf{F_{m\text{-}nanostickers}} + \mathbf{F_{intermolecule}}$; and $\mathbf{G_{nanosticker}}$ is formulated as $|\mathbf{G_{nanosticker}}| = m_{nanosticker} g = 9.8 m_{nanosticker}$ (N).

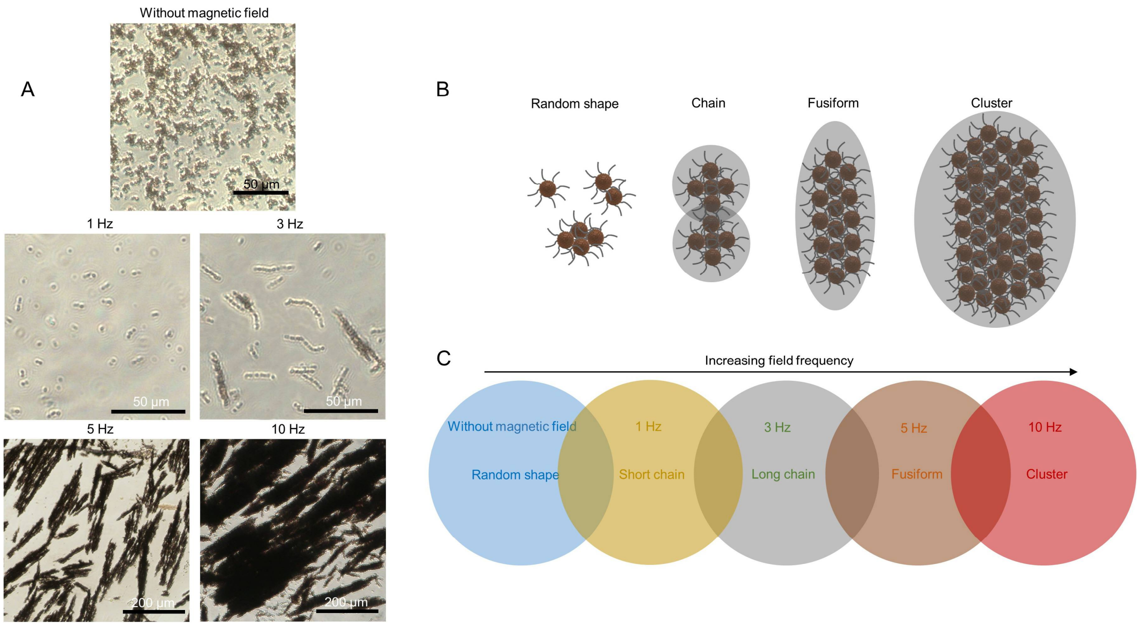

Supplementary Fig. 17. Nanostickers are assembled into different aggregation morphology upon different rotating frequency of magnetic field. (A) Optical microscope shows that nanostickers are randomly dispersed without magnetic field, while the morphology varies with the increased rotating frequency upon magnetic steering. (B) Schematic illustration for the aggregation morphology by the assembly of nanostickers. (C) Process diagram describes the variation tendency of aggregation morphology as the rotating frequency is increased.

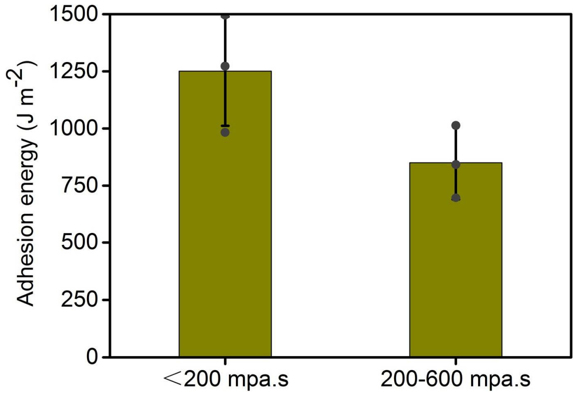

Supplementary Fig. 18. Comparison of adhesion energy induced by nanostickers with different viscosity. High-viscosity nanostickers generate larger hindrance than low-viscosity nanostickers upon magnetic control. Data are presented as mean ± SD; n = 3 independent samples.

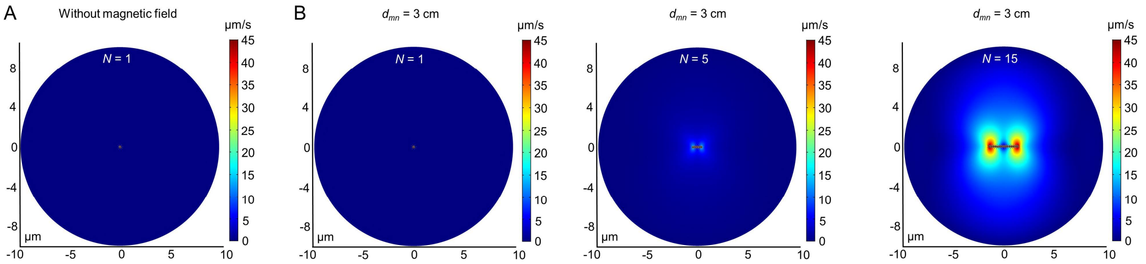

Supplementary Fig. 19. Simulation for the motion of nanostickers without and with magnetic field. (A) An individual nanosticker without magnetic control can produce negligible velocity. (B) Flow rate is largely improved with the increased chain length of assembled nanostickers.

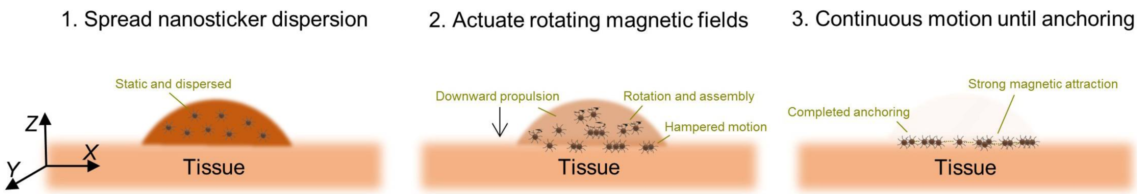

Supplementary Fig. 20. Schematic diagram for the anchoring procedure of nanostickers: 1. Spread nanosticker dispersion by different methods such as spraying, dropping, and brushing. 2. Actuate the rotating magnetic field to rotate nanostickers for assembly (*XY* plane) and propel nanostickers for bonding with tissues (*Z*-axis direction). 3. Nanostickers are successfully anchored when their motion is hampered by the rough tissues.

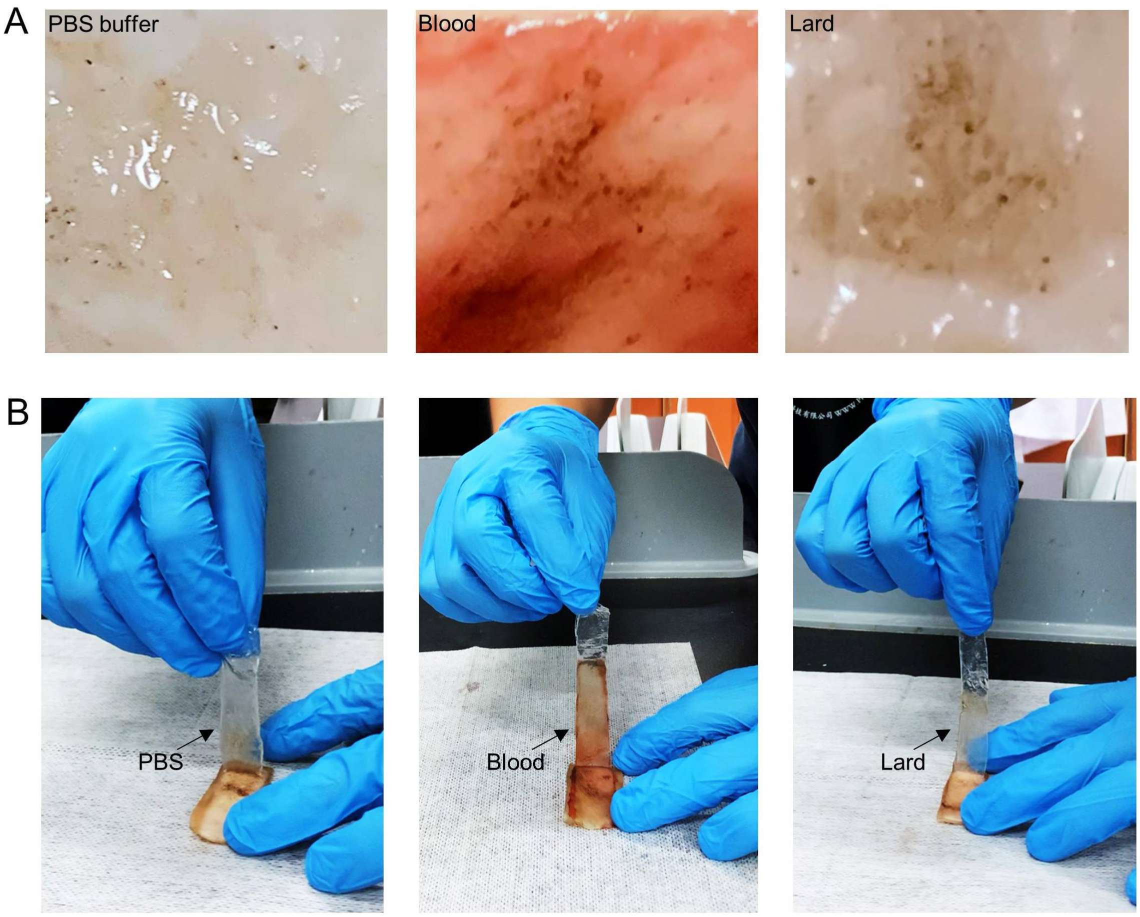

Supplementary Fig. 21. Propelling nanostickers to anchor on biofluid-contaminated pig skins. (A) Images show that nanostickers can pass through the biofluids such as PBS, blood, and lard to anchor on the skin. (B) Peeling of the patch displays that the skin-patch interfaces can be bridged by nanostickers even the skins are contaminated.

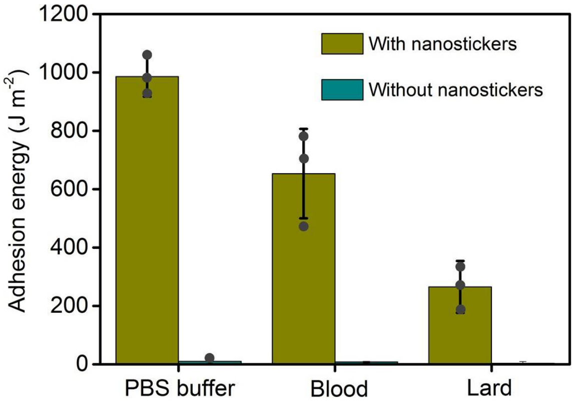

Supplementary Fig. 22. Measured adhesion energy on different biofluid-contaminated pig skins. Nanostickers can still be well anchored on biofluid-contaminated skins for patch attachment. Data are shown as mean ± SD; $n = 3$ independent samples.

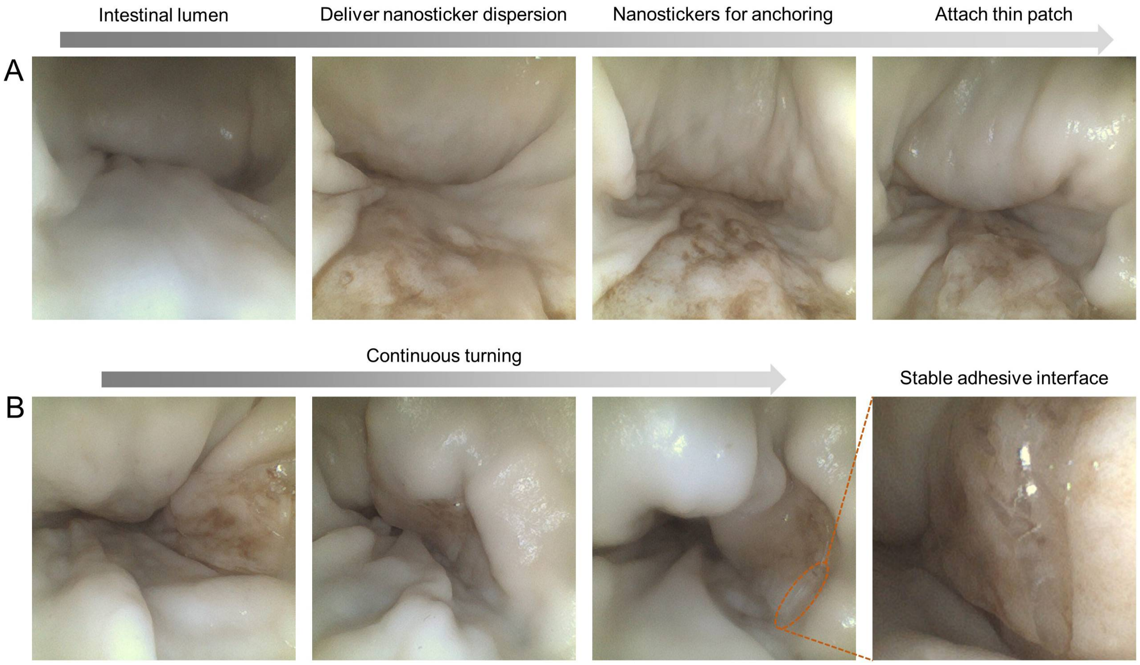

Supplementary Fig. 23. Images show the procedures of magnetic control bioadhesion in intestinal lumen. (A) Nanostickers are delivered into the target region for anchoring and then the thin patch can be attached. (B) The attached patch shows a conformal and stable adhesive interface with the intestinal lumen to bear with the mechanical movement.

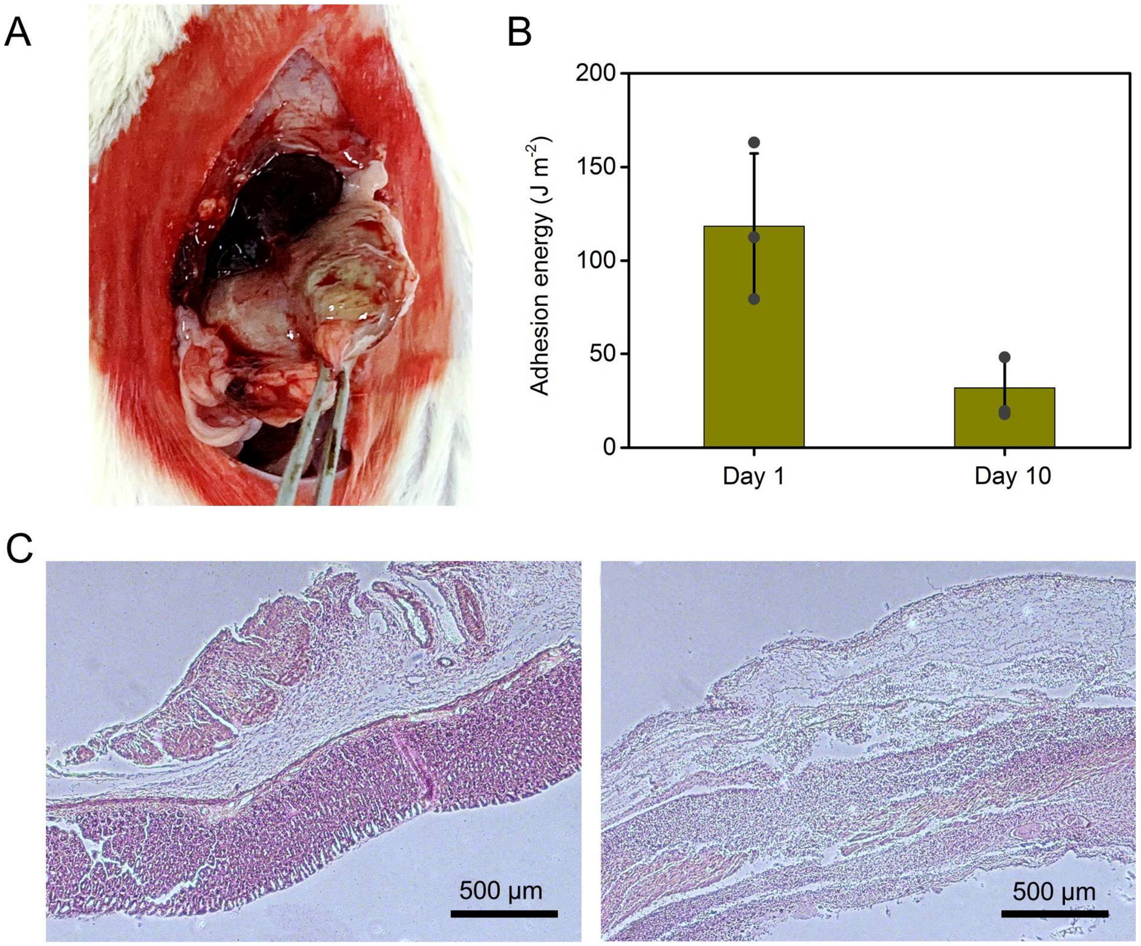

Supplementary Fig. 24. Adhesion investigations in vivo. Despite the adhesion energy is decayed after 10 days, it is still applicable as the patch should have been infiltrated by the tissues and integrated together. (A) The patch is attached on the anchored stomach surface and implanted in abdomen, which remains good adhesion after 10 days. (B) Adhesion energy measurement on different days after implantation in vivo. Data are shown as mean ± SD; *n* = 3 independent samples. (C) Histological analysis indicates that the nanostickers-anchored patch generates few stimuli to the underlying tissues (Left: normal group; Right: patched group).

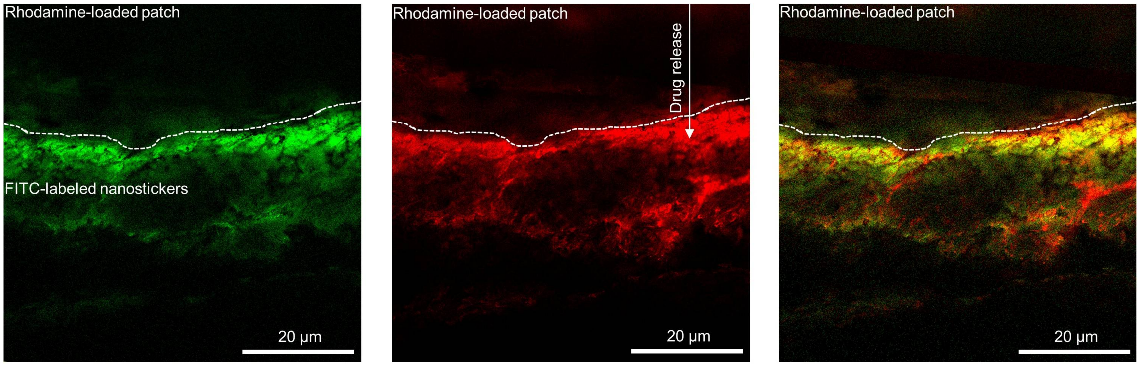

Supplementary Fig. 25. Drug release investigations after 24 h of attachment. FITC-labeled nanostickers are anchored on the tissue, showing bright green fluorescence. Meanwhile, rhodamine-loaded patch shows that most rhodamine (red fluorescence) is released into the tissue, as suggested by the colocalization of nanostickers and rhodamine. (FITC: excitation at 495 nm; rhodamine 6G: excitation at 550 nm)

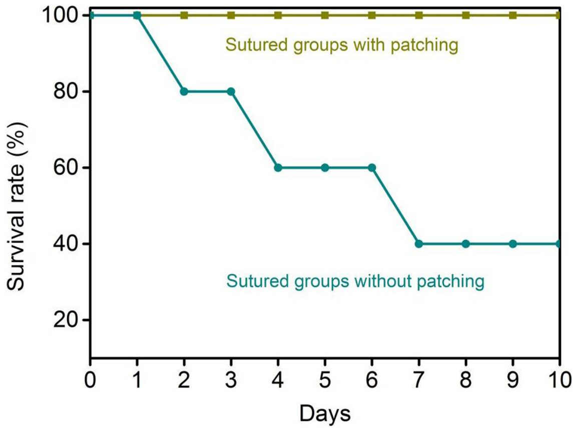

Supplementary Fig. 26. Survival rate comparison in different sutured groups. The 100% survival rate in patched groups indicates that the anchored patch can protect the injured intestines from serious complications to improve the survival rate.

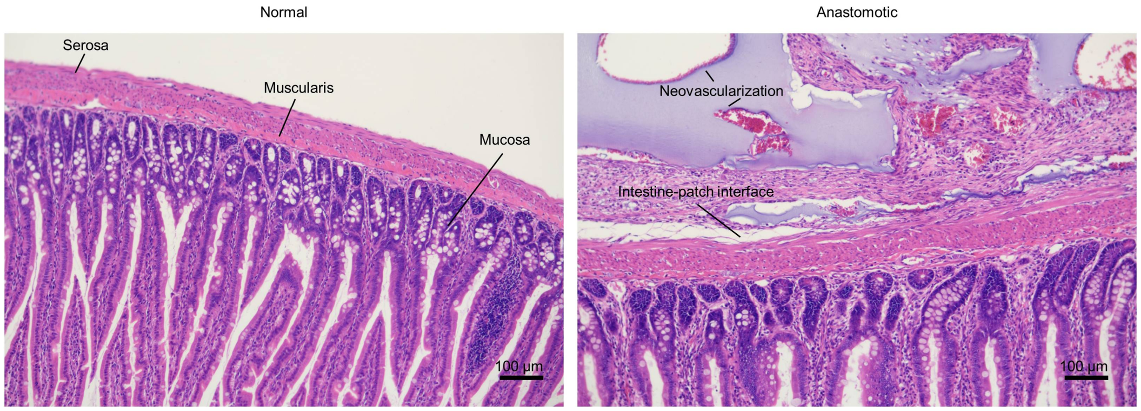

Supplementary Fig. 27. Histological analysis of enlarged images of the normal and patched groups. The patched group shows the structure of repaired intestines is similar to that of normal group, and the patch has been well infiltrated by tissues for 10 days of integration.

	Adhesion strategies	Adhesion mechanisms	Pro (s) in adhesion	Con (s) in adhesion
Conventional methods	Adhesive bonds	Chemical anchor	High adhesion energy; Available under complex interfacial environment (*e.g.*, biofluids)	Permanent and irreversible bonding; Uncontrollable spatiotemporally; Moderate fatigue resistance
		Non-covalent bond	Temporary and reversible bonding; Abundant designs for interfacial interactions (*e.g.*, hydrogen bonding, charge interaction)	Low adhesion energy; Easy bonding failure under complex interfacial environment (*e.g.*, biofluids); Poor fatigue resistance; Uncontrollable spatiotemporally
	Topological connection	Topological interlocking<br>Physical entanglement<br>Mechanical interlock	Adhesive properties (*e.g.*, adhesion energy, reversibility) are tunable by customized designs; Stimuli-responsive adhesion (*e.g.*, UV, pH, temperature) is achievable; Available under complex physiological environment (*e.g.*, biofluids)	Require complex and time-consuming processing on biological surfaces; Uncontrollable spatiotemporally in high level; Moderate fatigue resistance
Emerging Methods	Ultrasound mediation	US-induced cavitation	High adhesion energy; Strong fatigue resistance; Precise control in space and time; Applicable to diverse hydrogels and anchoring agents	Unsuitable for fragile body parts (*e.g.*, diseased region) and deep tissues due to the thick ultrasound probe and high pressure exerted by the probe; Acute thermal effects and potential damages under high power of treatment
	Magnetic control	Magnetic field gradient and vortex	High adhesion energy with minimum anchoring agents; Strong fatigue resistance; Remote control in adhesive properties (*e.g.*, adhesion energy, adhesion-related space and time); Applicable to diverse patches and anchoring agents; Harmless and applicable to a wide range of body parts	Non-uniform distribution of nanostickers due to magnetic field gradient

Supplementary Table 1. Overview of different methods and strategies for adhesion on biological tissues. Advantages and disadvantages are detailed for different methods and strategies to show the advancement of magnetic control bioadhesion.

Nanoparticle adhesive	Mass of nanoparticles in dispersion for bioadhesion	Adhesion energy generated from per milligram	Reference
2 wt%, ChsNCs	4-6 mg/200-300 μL	83.3-125 $J\ m^{-2}\ mg^{-1}$	*Science* **377**, 751-755 (2022)
2 wt%, CNC-CHO	4-6 mg/200-300 μL	30-45 $J\ m^{-2}\ mg^{-1}$	*Science* **377**, 751-755 (2022)
40 wt%, TSN	6 mg/15 μL	~1.7 $J\ m^{-2}\ mg^{-1}$	*Nat. Commun.* **8**, 15807 (2017)
30 wt%, AL-30	4.5 mg/15 μL	~0.36 $J\ m^{-2}\ mg^{-1}$	*Nature* **505**, 382-385 (2014)
20 wt%, ANP	~20 mg/~100 μL	~60 $J\ m^{-2}\ mg^{-1}$	*Nat. Commun.* **14**, 5378 (2023)
1 wt%, Nanostickers	1 mg/100 μL	~625 $J\ m^{-2}\ mg^{-1}$	/

Supplementary Table 2. Comparison of the adhesive performance between different nanoparticle-based adhesives. Compared to other representative nanoparticle-based adhesives, magnetic control method enables nanostickers to have a high adhesion energy at an ultralow dosage.

Supplementary References

1 Deruelle, M., Tirrell, M., Marciano, Y., Hervet, H. & Léger, L. Adhesion energy between polymer networks and solid surfaces modified by polymer attachment. *Faraday Discuss.* **98**, 55-65 (1994).

2 Yang, Z., Yang, H., Cao, Y., Cui, Y. & Zhang, L. Magnetically Actuated Continuum Medical Robots: A Review. *Adv. Intell. Syst.* **5**, 2200416 (2023).

3 Boyer, T. H. The force on a magnetic dipole. *Am. J. Phys.* **56**, 688-692 (1988).
